# Decreased Serum Sirtuin-1 in COPD

**DOI:** 10.1016/j.chest.2017.05.004

**Published:** 2017-08

**Authors:** Satoru Yanagisawa, Andriana I. Papaioannou, Anastasia Papaporfyriou, Jonathan R. Baker, Chaitanya Vuppusetty, Stelios Loukides, Peter J. Barnes, Kazuhiro Ito

**Affiliations:** a Airway Disease Section, National Heart and Lung Institute, Imperial College London, London, England; b Third Respiratory Medicine Department, Sismanogleion General Hospital, Athens, Greece; c First Respiratory Medicine Department, University of Athens, Sotiria Hospital, Athens, Greece; d Division of Respiratory Diseases I, National and Kapodistrian University of Athens, Athens, Greece

**Keywords:** biomarker, COPD, emphysema, serum, sirtuin-1, 6MWD, 6-min walking distance, Aado_2_, alveolar-arterial oxygen difference, BODE, BMI, airflow obstruction, dyspnea, and exercise capacity, Dlco, diffusing capacity for carbon monoxide, DMEM, Dulbecco's Modified Eagle Medium, ELISA, enzyme-linked immunosorbent assay, FBS, fetal bovine serum, GOLD, Global Initiative for Chronic Obstructive Lung Disease, HRCT, high-resolution CT, IC, inspiratory capacity, K_CO_%, diffusing capacity of the lung per unit volume, MRC, Medical Research Council, NAD, nicotinamide adenine dinucleotide, RPMI, Roswell Park Memorial Institute medium, RV, residual volume, s120S, serum SIRT1, SIRT1, silent information regulator 2 homolog 1, TLC, total lung capacity

## Abstract

**Background:**

The protein deacetylase sirtuin-1 (SIRT1) is an antiaging molecule that is decreased in the lung in patients with COPD. Recently, SIRT1 was reported to be detectable in serum, but serum SIRT1 (s120S) levels have not yet been reported in patients with COPD.

**Methods:**

Serum SIRT1 protein of all samples was measured by Western blot, and the SIRT1 protein band densities were calculated and compared with clinical parameters.

**Results:**

Several molecular sizes of SIRT1, including 120 kDa (actual size) and fragments (102 and 75 kDa) were quantified by Western blot. Among them, only the 120-kDa s120S was significantly decreased in patients with COPD compared with the control subjects without COPD (s120S ratio in healthy subjects = 0.90 ± 0.34 vs those with COPD = 0.68 ± 0.24; *P* = .014) and was positively correlated with airway obstruction (FEV_1_/FVC, *r* = 0.31*; P* = .020); its severity measured by FEV_1_ % predicted (*r* = 0.29*; P* = .029). s120S also showed a positive correlation with BMI (*r* = 0.36*; P* = .0077) and diffusing capacity of the lung per unit volume (the carbon monoxide transfer coefficient: K_CO_%) (*r* = 0.32*; P* = .025). It was also significantly decreased with increasing severity of lung emphysema (*r* = –0.40*; P* = .027) and with a clinical history of frequent COPD exacerbations (infrequent vs frequent, 0.76 ± 0.20 vs 0.56 ± 0.26*; P* = .027). SIRT1 was not detected in supernatant of A549 and primary epithelial cells in normal culture conditions.

**Conclusions:**

s120S was decreased in the patients with COPD, potentially as reflected by the reduced SIRT1 within cells as a result of oxidative stress, and might be a potential biomarker for certain disease characteristics of COPD.

Sirtuin-1 (*SIRT1*) is the mammalian homolog of silent information regulator (Sir2) family, initially described in yeast,^1^ and this highly preserved gene encodes nicotinamide adenine dinucleotide (NAD)-dependent protein deacetylases.^2^ Through modulating acetylating/deacetylating balances of multiple substrate proteins, SIRT1 regulates various cellular responses such as apoptosis, cellular senescence, endocrine metabolism, glucose homeostasis, and aging.^2^, ^3^, ^4^, ^5^, ^6^, ^7^ Although SIRT1 was originally described as a nuclear protein,^8^, ^9^ it has recently been shown that SIRT1 shuttles between the nucleus and cytoplasm,^10^, ^11^, ^12^ where it may associate with different target proteins in responding to divergent extracellular stimuli.^13^, ^14^, ^15^ Interestingly, SIRT1 has recently been measured in the serum,^16^ although its precise origin is unknown. In previous reports, serum SIRT1 (s120S) was consistently decreased with aging,^17^ and there was an accelerated reduction of serum SIRT1 in neurologic disorders such as Alzheimer’s disease,^16^ as well as in frailty^18^ and obesity,^19^, ^20^ all of which suggest that serum SIRT1 may be a potential biomarker for various aging-associated diseases. By contrast, an increase in serum SIRT1 has been reported in patients with asthma.^21^ However, the measurement of serum SIRT1 in other pulmonary diseases has not yet been elucidated.

COPD is a major global health problem.^22^, ^23^ In contrast to asthma, COPD is mainly caused by noxious gases such as cigarette smoke^24^, ^25^ and is characterized by poorly reversible small airway obstruction, emphysema, and corticosteroid-insensitive inflammation.^26^ COPD progresses slowly; therefore, most patients are elderly, and there is increasing evidence that it reflects accelerated aging of the lungs.^27^, ^28^, ^29^ SIRT1 is decreased in the peripheral lung and peripheral blood mononuclear cells in patients with COPD.^30^ In this study, we measured the serum levels of SIRT1 by Western blot in patients with COPD and age-matched control subjects and examined how it relates to characteristics of the disease.

## Methods

### Reagents

Commercially available reagents were obtained as follows: Roswell Park Memorial Institute medium (RPMI) medium 1640 (RPMI 1640) (No. 32404-014) and Dulbecco's Modified Eagle Medium (DMEM) (31053-028) were obtained from Life Technologies; fetal bovine serum (FBS), complete protease inhibitor cocktail (11836153001), and rabbit-derived anti-SIRT1 antibody (No. 5322) were obtained from Sigma-Aldrich; anti-β-actin antibody (ab6276) was obtained from Abcam; and goat-derived, peroxidase-conjugated antimouse (P0447) or antirabbit (P0448) secondary antibodies were obtained from DAKO.

### Patients and Healthy Volunteers for Serum

This project was approved by the Ethics Committee of Sismanogleio General Hospital (approval No. 5210-07/03/2012), and written informed consent was obtained from patients and healthy volunteers. COPD was defined and categorized according to the Global Initiative for Chronic Obstructive Lung Disease (GOLD) guidelines.^22^ Blood samples were taken from never smoker healthy subjects with normal lung function (NS, 12 subjects), smokers without COPD (SM, 19 subjects), and 26 patients with mild to very severe COPD (stages 1-2, 13 subjects; stages 3-4, 13 subjects) (Table 1). All patients with COPD were considered to be clinically stable, because none had required a change in their regular therapy during the 8 weeks preceding the sampling nor had they been treated with systemic corticosteroids or antibiotics. Patients with asthma, pneumonia, or lung cancer were excluded from the study. The smoking history of each subject was represented by the mean number of pack-years of cigarette consumption by ex-smokers and current smokers. All patients with COPD had a history of smoking, but all patients were asked to refrain from smoking for 3 hours before the serum sampling. Emphysema was characterized by high-resolution CT (HRCT).^31^ The degree of emphysema was determined using a visual emphysema score, as previously described.^32^ Briefly, emphysema was identified as areas of hypovascular low attenuation and was graded with a five-point scale based on the percentage of lung involved: 0 = no emphysema, 1 = up to 25% of the lung parenchyma involved, 2 = between 26% and 50% of lung parenchyma involved, 3 = between 51% and 75% of the lung parenchyma involved, and 4 = between 76% and 100% of lung parenchyma involved. Grades of the axial images of each lung were added and divided by the number of images evaluated to yield emphysema scores that ranged from 0 to 4. Patients with COPD were characterized as having frequent exacerbations if there were two or more severe exacerbations in 1 year.^33^ The Medical Research Council (MRC) dyspnea scale,^34^ Borg scale (dyspnea and fatigue),^35^ 6-min minute walking distance (6MWD),^36^ BMI, airflow obstruction, dyspnea, exercise capacity (BODE) index,^37^ and the Charlson index^38^ were examined according to the original reports. We also examined the air trapping by residual volume (RV)/total lung capacity (TLC) and the oxygenation capacity of lung by Pao_2_/Fio_2_ or by the alveolar-arterial oxygen difference (Aado_2_).

**Table 1:** Characteristics of Study Subjects

Variable	Nonsmokers	Smokers Without COPD	COPD Stages 1-2	COPD Stages 3-4
No. (M/F)	12 (3/9)	19 (11/8)	13 (10/3)	13 (11/2)
Age, y	65.3 ± 11.1	58.9 ± 12.3	64.6 ± 10.1	64.2 ± 11.1
Pack-years	0	38.9 ± 25.3	70.9 ± 27.2^a^	68.1 ± 25.8^a^
Emphysema	1 of 12	6 of 19	12 of 13	13 of 13
Emphysema score	0.29 ± 1.01	0.39 ± 0.74	1.33 ± 1.17	2.25 ± 1.20^a^^,^^b^
MRC dyspnea score	0.33 ± 0.89	0.68 ± 0.95	1.46 ± 0.78^c^	2.38 ± 1.12^a^^,^^b^
Charlson index	0.58 ± 1.00	0.95 ± 1.08	1.54 ± 1.27	1.92 ± 1.12^c^
Pao_2_ (mm Hg)	81.2 ± 7.2	77.7 ± 5.8	73.6 ± 6.9	66.7 ± 9.0^a^^,^^b^
Paco_2_ (mm Hg)	38.3 ± 2.4	39.5 ± 1.7	39.6 ± 2.7	39.8 ± 7.4
Pao_2_/Fio_2_ (mm Hg/%)	386.7 ± 34.4	370.2 ± 27.8	350.5 ± 33.0	307.7 ± 55.5^a^^,^^b^
Aado_2_	20.6 ± 9.4	22.6 ± 6.3	25.4 ± 7.2	41.0 ± 23.1^a^^,^^b^
Pao_2_/Paco_2_	2.12 ± 0.15	1.97 ± 0.17	1.82 ± 0.19^b^	1.72 ± 0.33^b^^,^^d^
BMI (kg/m^2^)	25.8 ± 3.2	29.0 ± 7.5	24.8 ± 3.6	23.0 ± 3.5^d^
FEV_1_ % predicted (%)	89.4 ± 12.6	89.7 ± 14.4	73.1 ± 14.6^a^^,^^c^	32.2 ± 7.8^a^^,^^b^
FVC % predicted (%)	84.2 ± 11.3	87.2 ± 14.9	90.3 ± 18.9	57.2 ± 11.7^a^^,^^b^
FEV_1_/FVC	84.4 ± 5.6	81.8 ± 9.2	61.4 ± 7.2^a^^,^^b^	45.9 ± 7.8^a^^,^^b^
Dlco % predicted (%)	78.9 ± 19.7	77.3 ± 17.3	63.5 ± 20.8	43.0 ± 13.7^a^^,^^b^
K_CO_ % predicted (%)	85.1 ± 16.9	89.2 ± 10.9	67.8 ± 19.5^a^	59.9 ± 14.0^a^^,^^b^
TLC % predicted (%)	83.7 ± 9.4	86.0 ± 25.6	94.6 ± 13.4	102.7 ± 36.2
FRC % predicted (%)	81.7 ± 8.3	82.2 ± 24.6	106.0 ± 27.1	110.3 ± 25.6^c^^,^^d^
RV % predicted (%)	82.8 ± 8.6	80.2 ± 20.7	107.9 ± 23.9^a^^,^^c^	120.6 ± 18.5^a^^,^^b^
RV/TLC	0.365 ± 0.062	0.324 ± 0.075	0.401 ± 0.085	0.473 ± 0.085^a^^,^^b^
IC % predicted (%)	84.8 ± 10.1	86.6 ± 23.8	85.5 ± 16.3	56.7 ± 16.5^a^^,^^b^
6MWD (m)	480.2 ± 110.2	508.6 ± 108.5	439.2 ± 122.4	359.6 ± 147.4^a^
DBOrgDyspnea	0.92 ± 2.27	0.68 ± 1.70	2.00 ± 1.73	3.15 ± 1.91^a^^,^^c^
DBorgFatigue	0.92 ± 1.68	0.68 ± 1.11	1.23 ± 1.17	2.38 ± 1.66^a^
Dsat (%)	–1.2 ± 4.4	–0.7 ± 3.2	–2.5 ± 4.6	–7.2 ± 4.7^a^^,^^b^
DHR	30.6 ± 8.3	21.3 ± 9.5	32.5 ± 23.1	22.3 ± 15.4
LABA/ LAMA/ ICS	1/ 1/ 0	2/ 2/ 3	8/ 9/ 3	10/ 10/ 10
CVD/ DM	2/ 2	7/ 2	6/ 1	5/ 3

Data are expressed as mean values ± SD. Pack-year represents the number of cigarettes smoked per day/20 × duration of smoking in years. Patients with COPD were categorized by the Global Initiative for Chronic Obstructive Lung Disease definition guidelines.^22^ Emphysematous phenotype was characterized according to the presence of significant emphysematous lesions (> 15% of lung parenchyma) by high-resolution CT.^31^ Patients with COPD were characterized as having frequent exacerbations if there were two or more exacerbations in 1 year.^33^

6MWD = six-min walking distance; Aado_2_ = alveolar-arterial oxygen difference; CVD = cardiovascular disease; DHR = difference in heart rate during 6MWD; DBorgDyspnea = the difference in Dyspnea level according to Borg scale between the end and the beginning of the 6-min walk test; DBorgFatigue = the difference in Fatigue level according to Borg scale between the end and the beginning of the 6-min walk test; Dlco = diffusing capacity of lung for carbon monoxide; Dsat = Desaturation on movement; DM = diabetes mellitus; FRC = functional residual capacity; IC = inspiratory capacity; ICS = inhaled corticosteroids; K_CO_ = the carbon monoxide transfer coefficient; LABA = long acting beta-agonists; LAMA = long-acting muscarinic antagonists; MRC = Medical Research Council; RV = residual volume; TLC = total lung capacity.

a *P* < .01 vs smokers without COPD.

b *P* < .01 vs nonsmokers.

c *P* < .05 vs nonsmokers.

d *P* < .05 vs smokers without COPD.

### Blood Sampling

Blood samples were collected in BD Vacutainer Plus Plastic Serum and SST Tubes, which are coated with silicone and micronized silica particles to accelerate clotting. Samples were then centrifuged at 1500*g* for 15 min at room temperature, and supernatants were aliquoted as serum samples and immediately stored at –70 ^o^C until measurement.

### Pulmonary Function Tests

Pulmonary function tests were performed using MasterScreen (Erich Jaeger GmbH) and included postbronchodilator FEV_1_, FVC, FEV_1_/FVC ratio, TLC, RV, inspiratory capacity (IC), and diffusing capacity for carbon monoxide (Dlco). Dlco and diffusing capacity for carbon monoxide adjusted for alveolar volume (DL_CO_/V_A_ or K_CO_) were assessed by the single-breath method with the patient in the sitting position. Lung function measurements were expressed as percentage of predicted values. Tests were performed according to the American Thoracic Society/European Respiratory Society guidelines by the same technician to ensure consistency of results. All lung function data are shown in Table 1.

### Serum SIRT1

Serum samples were diluted in radioimmunoprecipitation assay buffer (Sigma; 150 mM NaCl, 1.0% IGEPAL CA-630, 0.5% sodium deoxycholate, 0.1% sodium dodecyl sulfate, and 50 mM tris(hydroxymethyl)aminomethane, pH 8.0) completed with protease inhibitor, as previously published,^39^ separated by sodium dodecyl sulfate-polyacrylamide gel electrophoresis, transferred to nitrocellulose membrane, and incubated with anti-SIRT1 antibody or with anti-β-actin antibody overnight. The membranes were then incubated with the appropriate peroxidase-conjugated secondary antibodies. The bound antibodies were visualized by chemiluminescence (ECL Plus; GE Healthcare). The band density in each sample was normalized to the level of standard sample (from one healthy donor), and each SIRT1 protein level was shown as the relative ratio against that in standard sample.

### Cell Culture

BEAS-2B cells (SV40-immortalized human airway bronchial epithelial cell line) and A549 cells (human lung adenocarcinoma epithelial cell line) were purchased from the American Culture of Tissue Collection and grown in complete growth medium (RPMI 1640 and DMEM supplemented with heat-inactivated 10% FBS and 1% L-glutamine, respectively) at 37^o^C/5% CO_2_. Before use, cells were starved in minimum medium (RPMI 1640 or DMEM supplemented with 1% FBS and 1% L-glutamine), and cell culture supernatants were harvested at different time points. To eliminate the contamination of supernatant by free-floating cells, the supernatant was centrifuged and the upper half of the medium was taken as the sample.

Human primary bronchial epithelial cells obtained from three subjects without COPD and three subjects with COPD were cultured as monolayers in LHC-9 media (Invitrogen) on collagen (1% w/v)-coated plates as previously reported.^40^ Cells were extracted from lung tissue of patients undergoing lung resection at the Royal Brompton Hospital. All subjects gave informed written consent and the study was approved by the National Research Ethics Service London-Chelsea Research Ethics committee, study No. 09/H0801/85. All cells were serum starved 16 hours before stimulation. Cells were stimulated with 3% cigarette-smoke-conditioned media prepared as previously reported.^41^

### Statistical Analysis

Data from clinical samples were expressed as mean values ± SD. For the analysis of SIRT1, statistical significance was assessed using a nonparametric Kruskal-Wallis test with a Bonferroni multiple comparison procedure to exclude possible interaction between various variables within subgroups (Statcel 2, OMS Publishing Inc.). The analysis of correlation between each factor was performed by Spearman correlation coefficient rank sum test. All reported *P* values were two-sided, and *P* values < .05 were considered to be statistically significant.

## Results

**Figure 1:**
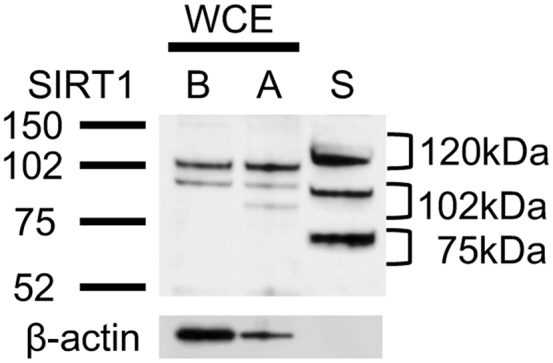
SIRT1 protein in serum. Western blot analysis of serum sample (S) was compared with whole-cell extracts of BEAS-2B cells (B) and A549 cells (A). SIRT1 = silent information regulator 2 homolog 1; WCE = whole cell extract.

**Figure 2:**
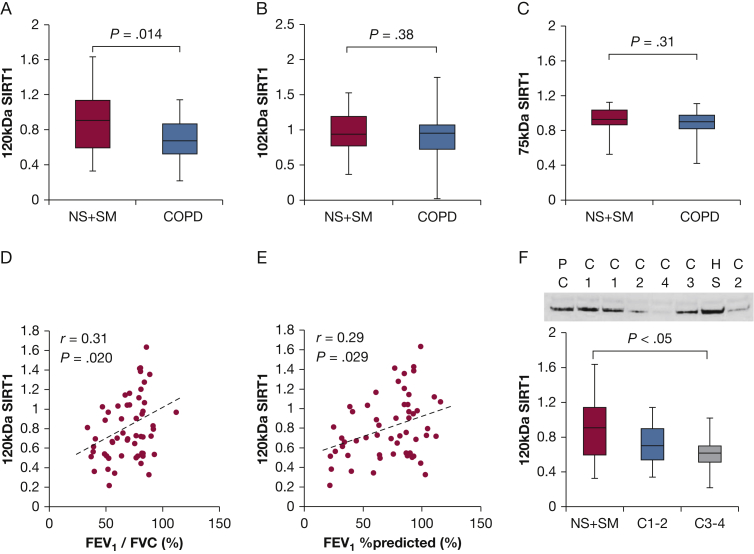
Reduced levels of 120-kDa serum SIRT1 (s120S) protein in COPD. A, s120S protein level in serum from healthy subjects and subjects with COPD (NS + SM) (C1-4 disease stage). Protein levels of (B) 102-kDa and (C) 75-kDa SIRT1 with or without COPD. D, Correlation between the s120S protein level and FEV_1_/FVC ratio. E, Correlation between the s120S protein level and FEV_1_ % predicted. F, s120S protein levels of healthy subjects and patients with COPD (NS + SM) of different stages (C1-2, C3-4). NS = nonsmoking subjects; PC = positive control from healthy subject; SM = smokers without COPD.

In a previous report, s120S was found to be detectable by Western blot,^18^ which showed an excellent correlation with the enzyme-linked immunosorbent assay (ELISA). As shown in Figure 1, anti-SIRT1 antibody used in this study detected different sizes of SIRT1, including 75, 102, and 120 kDa (the size originally reported) in BEAS-2B cells or A549 cells; therefore, we determined these SIRT1 fractions in serum samples separately. Compared with healthy subjects, the patients with COPD showed decreased levels of 120-kDa s120S (SIRT1 ratio in healthy subjects [NS + SM], 0.90 ± 0.34 vs subjects with COPD, 0.68 ± 0.24*; P* = .014) (Fig 2A), whereas SIRT1 with lower molecular weights (102 kDa and 75 kDa) did not (Figs 2B, 2C, e-Figs 1A, 1B, 1C). s120S showed a significant positive correlation with airway obstruction (FEV_1_/ FVC ratio; *r,* 0.31*; P* = .020) (Fig 2D, Table 2) and also with the severity of airway obstruction, measured by FEV_1_ % predicted (*r* = 0.29*; P* = .029) (Fig 2E), suggesting that s120S protein levels decrease with COPD progression (Fig 2F).

**Table 2:** Spearman Correlation Coefficient Rank Sum Test Between the s120S (120-kDa) SIRT1 and Patient Characteristics

Variable	All Subjects		Normal Subjects		COPD Only	
	*r*	*P* Value	*r*	*P* Value	*r*	*P* Value
BMI	0.36	.0077	0.28	.13	0.34	.092
Pack-year	–0.33	.014	–0.16	.38	–0.13	.51
FEV_1_/FVC	0.31	.021	–0.072	.69	0.32	.11
Emphysema score	–0.38	.0048	–0.082	.33	–0.34	.091
Kco % predicted	0.32	.025	0.059	.77	0.32	.13
FEV_1_ % predicted	0.29	.032	–0.088	.63	0.40	.046
Pao_2_/Paco_2_	0.28	.034	0.22	.22	0.058	.77
RV % predicted	–0.27	.054	0.11	.57	–0.23	.26
IC % predicted	0.28	.064	0.26	.20	0.25	.28
Dlco % predicted	0.26	.069	–0.043	.83	0.33	.10
Pao_2_/Fio_2_	0.23	.079	0.11	.55	0.036	.86
Pao_2_	0.22	.098	0.11	.55	–0.026	.90
6MWD	0.22	.11	–0.097	.60	0.45	.023
Dsat	0.20	.14	0.14	.45	0.12	.56
FVC % predicted	0.20	.14	–0.059	.75	0.27	.18
Aado_2_	–0.18	.17	–0.032	.86	–0.052	.80
FRC % predicted	–0.19	.18	0.10	.60	–0.083	.69
RV/TLC	–0.16	.24	–0.013	.95	–0.025	.90
Paco_2_	–0.06	.65	–0.097	.60	0.039	.85
TLC % predicted	–0.06	.66	0.21	.30	–0.10	.61

See Table 1 legend for expansion of abbreviations.

**Figure 3:**
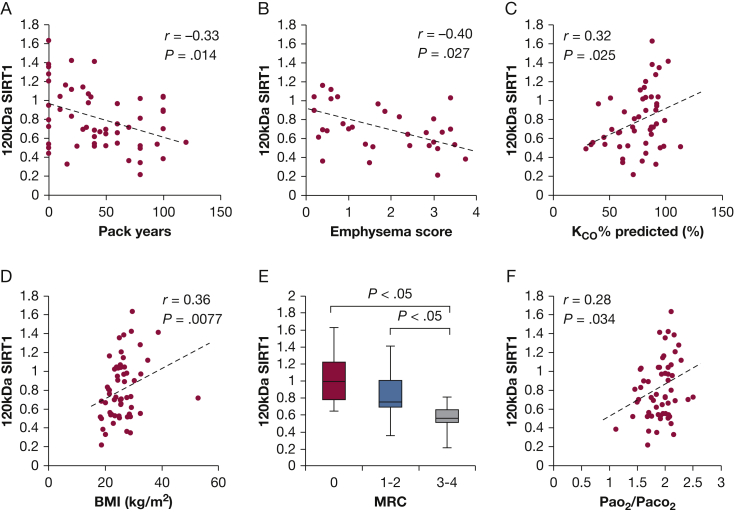
Serum SIRT1 (s120S) 120-kDa protein and patient characteristics. A, Relationship between the s120S protein level and cigarette smoke exposure in pack-years, (B) with emphysema score in all subjects demonstrating emphysema, (C) K_CO_ % predicted, (D) BMI, (E) MRC dyspnea score, and (F) Pao_2_/Paco_2_ ratio in all subjects. Pack-year represents the number of cigarettes smoked per day/20 × duration of smoking in years. K_CO_ = the carbon monoxide transfer coefficient; MRC = Medical Research Council.

In addition, s120S showed a negative correlation with the amount of cigarette consumption (pack-year; *r* = –0.33*; P* = .014) (Fig 3A). Patients with a higher degree of emphysema on HRCT had lower levels of s120S (*r* = –0.40*; P* = .027) (Fig 3B) when analyzed in all subjects showing some degree of emphysema. A good correlation was also observed in all subjects *(P* = .0048) (Table 2) and COPD subjects only *(P* = .091) (Table 2). In addition, patients with emphysema showed decreased levels of s120S when compared with the patients with normal lungs (SIRT1 ratio in control population, 0.92 ± 0.37 vs 0.71 ± 0.24 in those with emphysema; *P* = .026) (e-Fig 1D). This was confirmed by the significant positive correlation between the s120S SIRT1 and K_CO_ % predicted (*r* = 0.32*; P* = .025) (Fig 3C, Table 2). The s120S was not correlated with age, probably because of the elderly bias of samples included. In contrast, the BMI showed a significant positive correlation with s120S (*r* = 0.36*; P* = .0077) (Fig 3D, Table 2). In addition, s120S decreased significantly as symptoms (MRC dyspnea score) increased (Fig 3E). The severity of hypoxia (Pao_2_ or desaturation on movement) and oxygenation capacity of lung (Pao_2_/Fio_2_ or Aado_2_) did not show any correlation with s120S (Table 2); however, s120S showed positive correlation with Pao_2_/Paco_2_ ratio representing the combined effect on gas exchange^42^ (*r* = 0.28*; P* = .034) (Fig 3F), which suggested that the impairment of aerobic metabolism might contribute to the s120S protein level. Other patient background characteristics (Table 2) or subject comorbidities (such as cardiovascular disease or diabetes mellitus) and Charlson index did not show any association with serum levels of SIRT1.

**Figure 4:**
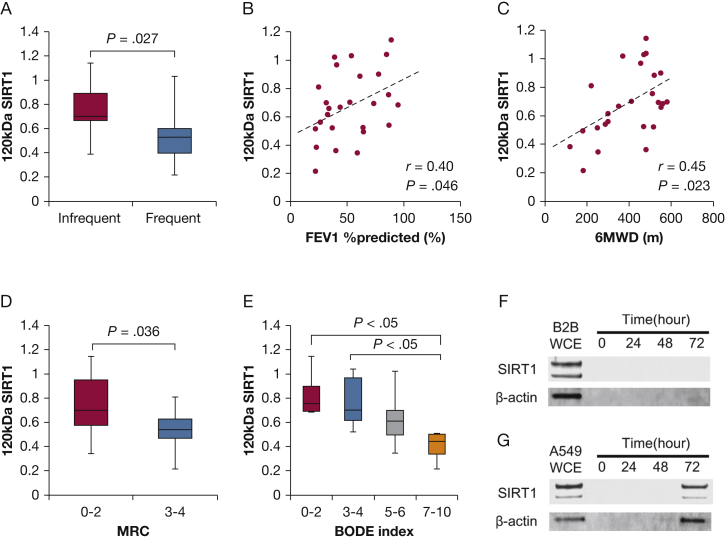
The s120S protein levels of patients with COPD. A, The s120S protein level in relation to frequent exacerbations of COPD. Relationship between the s120S protein level and (B) FEV_1_ % predicted, (C) 6MWD, (D) MRC dyspnea score, and (E) s120S protein levels in patients with COPD with different BODE index. F, Time dependency of SIRT1 in cell-culture supernatant from BEAS-2B cells (B2B) or (G) A549 cells. 6MWD = 6-min walking distance; BODE = BMI, airflow obstruction, dyspnea, and exercise capacity. See Figure 1 legend for expansion of other abbreviations.

When we limited analysis to the patients with COPD only, we identified two additional findings. First, patients with COPD with frequent exacerbations tend to have lower s120S levels compared with those with stable disease (Fig 4A). Second, s120S had a positive correlation not only with the FEV_1_ % predicted (*r* = 0.40*; P* = .046) (Fig 4B) but also with 6MWD (*r* = 0.45*; P* = 0.023) (Fig 4C). This was also confirmed by the fact that s120S was negatively associated with the MRC dyspnea score (Fig 4D) and with the BODE index (Fig 4E), which is known to be a strong predictor of long-term prognosis in COPD.^37^ We further analyzed the correlation with all parameters in the GOLD stage 1-2 population only and the 3-4 population only. We did not see any significant correlation except for IC % in stages 1-2 (e-Table 1).

Regarding the origin of s120S, we were unable to detect any active secretion from lung epithelial cell lines such as BEAS-2B (airway) cells or A549 (parenchymal) cells (Figs 4F, 4G). In A549 cells, SIRT1 was detected in supernatant after 72 hours of culturing, but as the housekeeping protein β-actin was also detected, it was likely that this may have been due to increased cell permeability related to loss of function. Furthermore, we also investigated SIRT1 protein release in supernatant from non-COPD (n = 3) and COPD (n = 3) primary bronchial epithelial cells. We did not find original or degraded SIRT1 protein or β-actin in supernatant in the absence or presence of cigarette-smoke-conditioned media (data not shown). Thus, it was unlikely that SIRT1 was excreted from bronchial epithelial cells.

## Discussion

In the current study, we showed for the first time, to our knowledge, that the protein levels of s120S in its 120-kDa form were significantly decreased in patients with COPD. The s120S protein levels were positively correlated with the severity of airway obstruction and showed a strong negative correlation with the amount of cigarette consumption, suggesting that oxidative stress may lead to the reduction of s120S. This is contrast to bronchial asthma,^21^ in which s120S detected by ELISA was increased. In addition, we found that s120S was significantly correlated with the severity of emphysema (HRCT reading and K_CO_ % predicted) and functional disability represented by an MRC dyspnea score, 6MWD, and BODE score. These results suggest that s120S may be a useful marker for assessing certain disease characteristics in patients with COPD.

Among the seven sirtuin isozymes,^43^ SIRT1 is the most widely studied in mammals from the viewpoint of regulation by oxidative stress, which is relevant to cellular senescence and chronic inflammation.^2^, ^3^, ^4^ In fact, dysregulation of SIRT1 has been described not only in aging-associated diseases but also in those associated with long-term cigarette smoking,^44^, ^45^ and all are characterized by oxidant/antioxidant imbalance.^46^ We previously reported a reduction in SIRT1 in the peripheral lungs of patients with COPD.^30^ Although reports of reduced SIRT1 relate to intracellular SIRT1 (mRNA or protein), Kumar et al^16^ first reported that SIRT1 was detectable in the serum. In this report, s120S was measured by various methods, including Western blot, ELISA, and surface plasmon resonance, with good correlation with each method, confirming that SIRT1 is a serum protein. Interestingly, they also showed significant reduction of s120S protein levels as dementia progressed, suggesting that s120S may be a useful biomarker for assessing cognitive disease. This report was surprising, as SIRT1 had originally been described only as a nuclear protein.^8^, ^9^ However, recent reports have demonstrated that SIRT1 can shuttle between the nucleus and cytoplasm^10^, ^11^, ^12^; therefore, SIRT1 is potentially present in the extracellular component.

The strength of our study is the selective determination of the fraction of full-length SIRT1 (120 kDa) separate from other truncated SIRT1 proteins by Western blot. This is in contrast to previous reports that used ELISA for s120S detection.^18^, ^19^, ^20^, ^21^ Despite its good quantitative capability, ELISA does not appear to be specific for the full-length functional fraction of SIRT1, because antibodies recognize several fractions with the target motif, irrespective of their function (Fig 1A). In previous reports, several bands of SIRT1 protein (original and truncated proteins) were described, indicating different molecular weights by Western blot,^47^, ^48^ each of which may function differently, although this has not yet been elucidated. Therefore, our results are unique, as we were able to analyze only the fully functional fraction of full-length SIRT1 (120-kDa SIRT1) that was separated by Western blot. Thus, Western blot should be used for s120S detection.

In addition, this is the first report to show that s120S is reduced in patients with COPD. Compared with healthy subjects, patients with COPD showed decreased levels of s120S, which correlated not only with the airway obstruction but also with its severity, resultant lung emphysema, and decreased diffusion capacity. These results were compatible with the previous reports that found that the SIRT1 protein level was decreased in peripheral lung or peripheral mononuclear cells in patients with COPD.^30^ Furthermore, we could also detect the association of s120S protein levels with BMI, MRC dyspnea score, and Pao_2_/Paco_2_ imbalance, all of which suggested that s120S is not just an indicator of lung damage but is a surrogate marker for oxygen metabolism and systemic metabolic status. Interestingly, our result appears to be opposite to that reported in patients with asthma^21^; therefore, s120S might be a potential biomarker to help to differentiate these two diseases. Future studies might be necessary for comparing the s120S levels directly between patients with asthma and COPD populations. Since reduced s120S is also reported in association with frailty in elderly people, it may also be useful in understanding the multimorbidity associated with COPD. We further analyzed the correlation with all parameters in a GOLD stage 1-2 population only and in a GOLD stage 3-4 population only. We did not see any significant correlation in all parameters except for IC % predicted in the stage 1-2 population. However, we observed a nearly significant correlation in FEV_1_/FVC *(P* = .19), emphysema score *(P* = .080), K_CO_ % *(P* = .17), RV % *(P* = .090), and 6MWD *(P* = .11) in the stage 1-2 population. We did not have enough power to demonstrate the association in the current study, but a future large study will reveal the usefulness of s120S as a potential biomarker to determine the early stage of COPD.

A limitation of the present study is that we have not identified the precise source of SIRT1 in serum. We could not detect any fractions of SIRT1 in the cell culture supernatant in A549 and BEAS-2B epithelial cells, indicating that cellular leakage or active secretion is unlikely. In primary bronchial epithelial cells, we could not find full or degraded SIRT1 proteins or β-actin in supernatant in the presence or absence of cigarette-smoke-conditioned media, suggesting that SIRT1 is unlikely secreted by bronchial epithelial cells. However, SIRT1 protein was detected in supernatant from A549 cells at a later stage, which was associated with an increase in β-actin expression. This suggests that epithelial cells are still a possible source of SIRT1 when cells are damaged. Considering that SIRT1 in patients with COPD has been reported to be decreased not only in the lung^30^ but also in endothelial progenitor cells^49^ and circulating leukocytes,^30^, ^50^ decreased s120S might reflect the reduction of SIRT1 in cells as a result of oxidative stress. Peripheral blood mononuclear cells or alveolar macrophages might be potential sources of SIRT1. It might be necessary in a future study to identify the precise origin of s120S and the factors that modulate s120S levels in COPD and other chronic aging diseases. Secondarily, even though there were statistical differences in SIRT1 levels between the comparison groups, there was significant overlap in the values of all groups. GOLD stage is defined by lung function (mainly by FEV_1_ % predicted), but, as discussed earlier, there was good correlation between SIRT1 and certain disease characteristics such as emphysema, MRC dyspnea score, 6MWD, and BODE score. Therefore, SIRT1 level is influenced by several factors rather than lung function alone. The current study is too small to evaluate further, but we believe that a future large study with more patients will provide a novel approach to classify disease stage based on SIRT1.

## Conclusions

In summary, we report for the first time that s120S was reduced in patients with COPD and that this reduction was correlated with the extent of emphysema and reduced functional measurements that correlate with disease progression. Serum SIRT1 might therefore serve as a potential biomarker for COPD.

## Supplementary Data

e-Figure 1 and e-Table 1

## References

[1] Imai S., Armstrong C.M., Kaeberlein M., Guarente L. (2000). Transcriptional silencing and longevity protein Sir2 is an NAD-dependent histone deacetylase. Nature.

[2] Hwang J.W., Yao H., Caito S., Sundar I.K., Rahman I. (2013). Redox regulation of SIRT1 in inflammation and cellular senescence. Free Radic Biol Med.

[3] Rahman S., Islam R. (2011). Mammalian Sirt1: insights on its biological functions. Cell Commun Signal.

[4] Salminen A., Kaarniranta K., Kauppinen A. (2013). Crosstalk between oxidative stress and SIRT1: impact on the aging process. Int J Mol Sci.

[5] Nogueiras R., Habegger K.M., Chaudhary N. (2012). Sirtuin 1 and sirtuin 3: physiological modulators of metabolism. Physiol Rev.

[6] Nakagawa T., Guarente L. (2011). Sirtuins at a glance. J Cell Sci.

[7] Houtkooper R.H., Pirinen E., Auwerx J. (2012). Sirtuins as regulators of metabolism and healthspan. Nat Rev Mol Cell Biol.

[8] McBurney M.W., Yang X., Jardine K. (2003). The mammalian SIR2alpha protein has a role in embryogenesis and gametogenesis. Mol Cell Biol.

[9] Sakamoto J., Miura T., Shimamoto K., Horio Y. (2004). Predominant expression of Sir2α, an NAD-dependent histone deacetylase, in the embryonic mouse heart and brain. FEBS Lett.

[10] Moynihan K.A., Grimm A.A., Plueger M.M. (2005). Increased dosage of mammalian Sir2 in pancreatic β cells enhances glucose-stimulated insulin secretion in mice. Cell Metab.

[11] Chen I.Y., Lypowy J., Pain J. (2006). Histone H2A.z is essential for cardiac myocyte hypertrophy but opposed by silent information regulator 2alpha. J Biol Chem.

[12] Stünkel W., Peh B.K., Tan Y.C. (2007). Function of the SIRTI protein deacetylase in cancer. Biotechnol J.

[13] Rosenberg M.I., Parkhurst S.M. (2002). Drosophila Sir2 is required for heterochromatic silencing and by euchromatic Hairy/E(Spl) bHLH repressors in segmentation and sex determination. Cell.

[14] Tanno M., Sakamoto J., Miura T., Shimamoto K., Horio Y. (2007). Nucleocytoplasmic shuttling of the NAD+-dependent histone deacetylase SIRT1. J Biol Chem.

[15] Meng C., Wu S., Xing D. (2011). Real-time fluorescence imaging of Sirt1 cytosolic translocation under the treatment of growth factor deprivation. J Innov Opt Health Sci.

[16] Kumar R., Chaterjee P., Sharma P.K. (2013). Sirtuin1: a promising serum protein marker for early detection of Alzheimer’s disease. PLoS One.

[17] Zhong Y., Chen A.F., Zhao J., Gu Y.J., Fu G.X. (2016). Serum levels of cathepsin D, sirtuin1, and endothelial nitric oxide synthase are correlatively reduced in elderly healthy people. Aging Clin Exp Res.

[18] Kumar R., Mohan N., Upadhyay A.D. (2014). Identification of serum sirtuins as novel noninvasive protein markers for frailty. Aging Cell.

[19] Caglayan E.K., Engin-Ustun Y., Gocmen A.Y. (2016). Is there any relationship between serum sirtuin-1 level and neutrophil-lymphocyte ratio in hyperemesis gravidarum?. J Perinat Med.

[20] Mariani S., Fiore D., Persichetti A. (2016). Circulating SIRT1 increases after intragastric balloon fat loss in obese patients. Obes Surg.

[21] Wang Y., Li D., Ma G. (2015). Increases in peripheral SIRT1: a new biological characteristic of asthma. Respirology.

[22] Global Initiative for Chronic Obstructive Lung Disease. Global strategy for the diagnosis, management, and prevention of chronic obstructive pulmonary disease, updated 2014. GOLD website. http://www.goldcopd.org. Accessed June 3, 2017.

[23] Postma D.S., Kerkhof M., Boezen H.M., Koppelman G.H. (2011). Asthma and chronic obstructive pulmonary disease: common genes, common environments?. Am J Respir Crit Care Med.

[24] DeMarco R., Accordini S., Marcon A. (2011). Risk factors for chronic obstructive pulmonary disease in a European cohort of young adults. Am J Respir Crit Care Med.

[25] Willemse B.W.M., Postma D.S., Timens W., ten Hacken N.H.T. (2004). The impact of smoking cessation on respiratory symptoms, lung function, airway hyperresponsiveness and inflammation. Eur Respir J.

[26] To Y., Ito K., Kizawa Y. (2010). Targeting phosphoinositide-3-kinase-δ with theophylline reverses corticosteroid insensitivity in chronic obstructive pulmonary disease. Am J Respir Crit Care Med.

[27] Ito K., Barnes P.J. (2009). COPD as a disease of accelerated lung aging. Chest.

[28] Ito K., Colley T., Mercado N. (2012). Geroprotectors as a novel therapeutic strategy for COPD, an accelerating aging disease. Int J COPD.

[29] Mercado N., Ito K., Barnes P.J. (2015). Accelerated ageing of the lung in COPD: new concepts. Thorax.

[30] Nakamaru Y., Vuppusetty C., Wada H. (2009). A protein deacetylase SIRT1 is a negative regulator of metalloproteinase-9. FASEB J.

[31] Boschetto P., Quintavalle S., Zeni E. (2006). Association between markers of emphysema and more severe chronic obstructive pulmonary disease. Thorax.

[32] Park K.J., Bergin C.J., Clausen J.L. (1999). Quantitation of emphysema with three-dimensional CT densitometry: comparison with two-dimensional analysis, visual emphysema scores, and pulmonary function test results. Radiology.

[33] Hurst J.R., Vestbo J., Anzueto A. (2010). Susceptibility to exacerbation in chronic obstructive pulmonary disease. N Engl J Med.

[34] Mahler D.A., Wells C.K. (1988). Evaluation of clinical methods for rating dyspnea. Chest.

[35] Borg G.A. (1982). Psychophysical bases of perceived exertion. Med Sci Sports Exerc.

[36] Crapo R.O., Casaburi R., Coates A.L. (2002). ATS statement: guidelines for the six-minute walk test. Am J Respir Crit Care Med.

[37] Celli BR, Cote CG, Marin JM, et al. The body-mass index, airflow obstruction, dyspnea, and exercise capacity index in chronic obstructive pulmonary disease. *N Engl J Med*. 2004;350(10):1005-1012. 10.1056/NEJMoa021322 14999112

[38] Charlson M.E., Pompei P., Ales K.L., MacKenzie C.R. (1987). A new method of classifying prognostic comorbidity in longitudinal studies: development and validation. J Chronic Dis.

[39] Ito K., Ito M., Elliott W.M. (2005). Decreased histone deacetylase activity in chronic obstructive pulmonary disease. N Engl J Med.

[40] Baker J.R., Vuppusetty C., Colley T. (2016). Oxidative stress dependent microRNA-34a activation via PI3Kalpha reduces the expression of sirtuin-1 and sirtuin-6 in epithelial cells. Sci Rep.

[41] Mercado N., Kizawa Y., Ueda K. (2014). Activation of transcription factor Nrf2 signalling by the sphingosine kinase inhibitor SKI-II is mediated by the formation of Keap1 dimers. PLoS One.

[42] Saaresranta T., Polo-Kantola P., Irjala K., Helenius H., Polo O. (1999). Respiratory insufficiency in postmenopausal women: sustained improvement of gas exchange with short-term medroxyprogesterone acetate. Chest.

[43] Frye R.A. (2000). Phylogenetic classification of prokaryotic and eukaryotic Sir2-like proteins. Biochem Biophys Res Commun.

[44] Stunkel W., Campbell R.M., Hdac T.M., Stunkel W., Campbell R.M. (2011). Sirtuin 1 (SIRT1): the misunderstood HDAC. J Biomol Screen.

[45] Cantó C., Sauve A.A., Bai P., Cantó C., Sauve A.A., Bai P. (2013). Crosstalk between poly(ADP-ribose) polymerase and sirtuin enzymes. Mol Aspects Med.

[46] Kwon H.S., Ott M. (2008). The ups and downs of SIRT1. Trends Biochem Sci.

[47] Oppenheimer H., Gabay O., Meir H. (2012). 75-Kd sirtuin 1 blocks tumor necrosis factor-α mediated apoptosis in human osteoarthritic chondrocytes. Arthritis Rheum.

[48] Tong C., Morrison A., Mattison S. (2013). Impaired SIRT1 nucleocytoplasmic shuttling in the senescent heart during ischemic stress. FASEB J.

[49] Paschalaki K.E., Starke R.D., Hu Y. (2013). Dysfunction of endothelial progenitor cells from smokers and chronic obstructive pulmonary disease patients due to increased DNA damage and senescence. Stem Cells.

[50] Rutten E.P.A., Gopal P., Wouters E.F.M. (2016). Various mechanistic pathways representing the aging process are altered in COPD. Chest.

