# Supplementary material for: Decreased Serum Sirtuin-1 in COPD
Source: Chest. 2017 Aug;152(2):343–52. doi: 10.1016/j.chest.2017.05.004 (PMC5540026; doi:10.1016/j.chest.2017.05.004)
Supplement: e-Figure 1 and e-Table 1 [file mmc1.pdf]

CHEST Online Supplement

Decreased Serum Sirtuin-1 in COPD

Satoru Yanagisawa, MD, PhD; Andriana I. Papaioannou, MD, PhD; Anastasia Papaporfyriou, MD, PhD; Jonathan R. Baker, PhD; Chaitanya Vuppusetty, MSc; Stelios Loukides, MD, PhD; Peter J. Barnes, DM, DSc; and Kazuhiro Ito, DVM, PhD

CHEST 2017; 152(2):343-352

Online supplements are not copyedited prior to posting and the author(s) take full responsibility for the accuracy of all data.

© 2017 AMERICAN COLLEGE OF CHEST PHYSICIANS. Reproduction of this article is prohibited without written permission from the American College of Chest Physicians. See online for more details. **DOI:** j.chest.2017.05.004

e-Figure 1

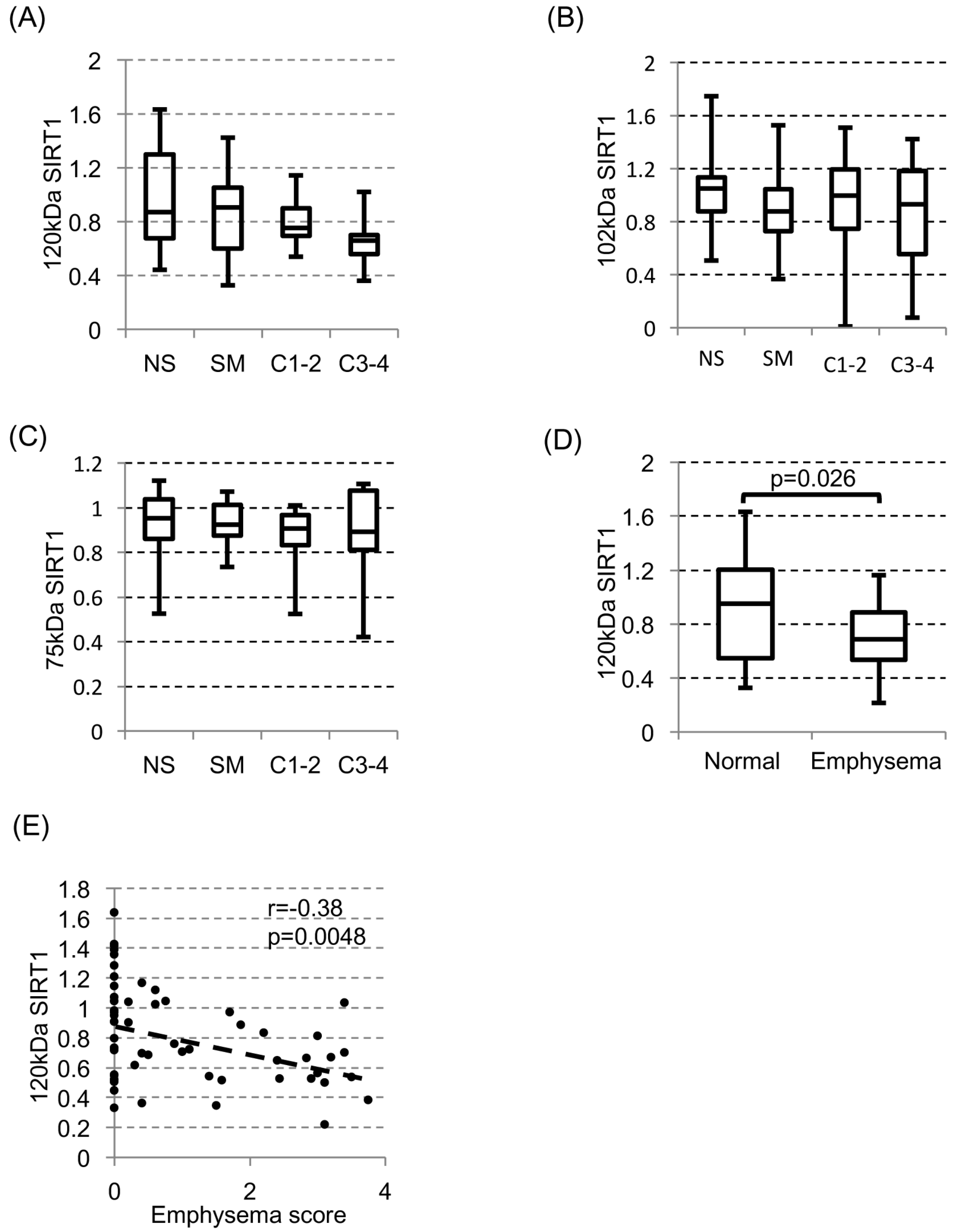

e-Figure 1. Reduced levels of serum 120kDa SIRT1 (s120S) protein in COPD and correlation with Emphysema. The levels of SIRT1 at 120 kDa (A), 102kDa (B) and 75kDa (C) in serum from healthy non-smoker subjects (NS), smokers without COPD (SM) and COPD patients (C1-2 or C3-4 disease stage). Comparison of SIRT1 120KDa between subjects with normal lung and with emphysema (D) and correlation between SIRT1 120KDa and emphysema score in all subjects (E).

Online supplements are not copyedited prior to posting and the author(s) take full responsibility for the accuracy of all data.

e-Table 1. The Spearman's correlation coefficient rank test between the serum SIRT1 (120kDa) and patient characteristics

	COPD Stage 1-2		COPD Stage 3-4	
	r	p	r	p
BMI	0.35	0.22	0.32	0.26
Pack-year	-0.070	0.81	-0.23	0.43
FEV_1/FVC	0.38	0.19	-0.0055	0.98
Emphysema Score	-0.51	0.080	-0.069	0.81
Kco % predicted	0.44	0.17	-0.14	0.62
FEV_1 % predicted	0.18	0.53	0.47	0.10
$PaO_2/PaCO_2$	-0.24	0.41	0.26	0.37
RV % predicted	-0.51	0.090	0.050	0.86
IC % predicted	-0.83	0.018	0.25	0.46
DLCO % predicted	0.27	0.38	0.19	0.52
PaO_2 / FiO_2	-0.25	0.40	0.094	0.74
PaO_2	-0.25	0.40	-0.072	0.80
6MWD	0.47	0.11	0.35	0.22
Dsat	-0.19	0.50	0.32	0.27
FVC % predicted	-0.099	0.73	0.41	0.16
$AaDO_2$	0.18	0.53	-0.14	0.62
FRC % predicted	-0.15	0.61	0.028	0.93
RV/TLC	-0.20	0.50	0.27	0.35
$PaCO_2$	0.16	0.59	-0.16	0.57
TLC % predicted	-0.12	0.68	-0.082	0.78

Abbreviations: r = correlation coefficient; p = probability value.

170106

Online supplements are not copyedited prior to posting and the author(s) take full responsibility for the accuracy of all data.
